# The Salts of Morphia

**Published:** 1851-03

**Authors:** 


### ARTICLE VIII.

### *The Salts of Morphia.*

M Mialhe, of Paris, is of the opinion that Opium, either in the shape of extract or tincture, ought to be entirely discarded from practice, as the proportion of active principles in this drug is extremely uncertain, both from natural causes, and through adulterations. He has found that in the various kinds of opium of commerce, morphia varies from seven grains and a half to eight scruples per three ounces and a half; or in other words, from one-half to ten per cent. In adulterated specimens, namely in a substance that merely imitated opium, he has found only six parts of morphia in 5000. M. Mialhe infers that morphia alone should be used in medicine, and that this principle should drive away opium, as quinine has replaced bark.—*London Lancet.*, *in ibid.*

